# Supplementary figures and images for: Socioeconomic status and the likelihood of informal care provision in Japan: An analysis considering survival probability of care recipients
Source: PLoS One. 2021 Aug 13;16(8):e0256107. doi: 10.1371/journal.pone.0256107 (PMC8362941; doi:10.1371/journal.pone.0256107)

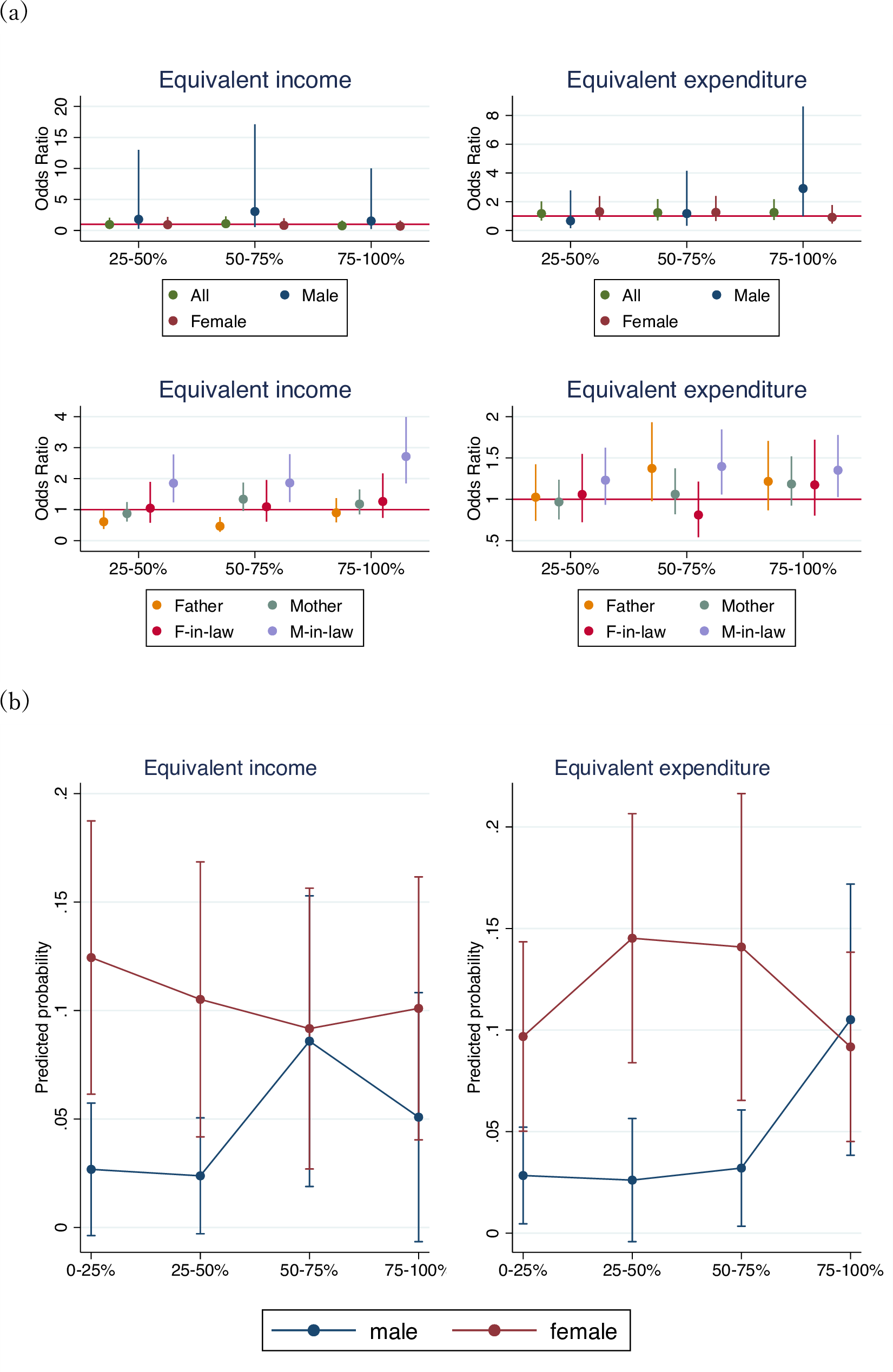

Supplement: S1 Fig — (a) Odds ratio of providing care and survival of parents; (b) care provision considering selection by survival. In the analysis in Figs 1–3, we use household income and monthly expenditure as SES measures. Although the information on household size is available only for the 2009 and 2011 surveys, we conduct the same analysis using equivalent income and expenditure, whereby household income and monthly expenditure are divided by the square root of household size. Panel (a) shows the odds ratio of providing care (top) and survival (bottom) based on logistic regressions. Panel (b) shows the predicted probability of care provision to parents using inverse probability weighting. In the first stage, age, age squared, gender, marital status of respondents, and year effects were included as dependent variables. In the second stage, the working status was also included. In both panels, the bars represent the 95% confidence interval. Each panel shows a different SES measure. The sample used for each analysis was the same as in the main analysis. (a) The number of observations for all the respondents in the top is 900 and 1,459 for equivalent income and expenditure, and the corresponding number of observations in the bottom ranges from 1,521 (parents-in-law for equivalent income) to 2,835 (parents for equivalent expenditure). (b) The number of observations is 900 for equivalent income and 1,459 for equivalent expenditure. (TIF) [file pone.0256107.s001.tif]

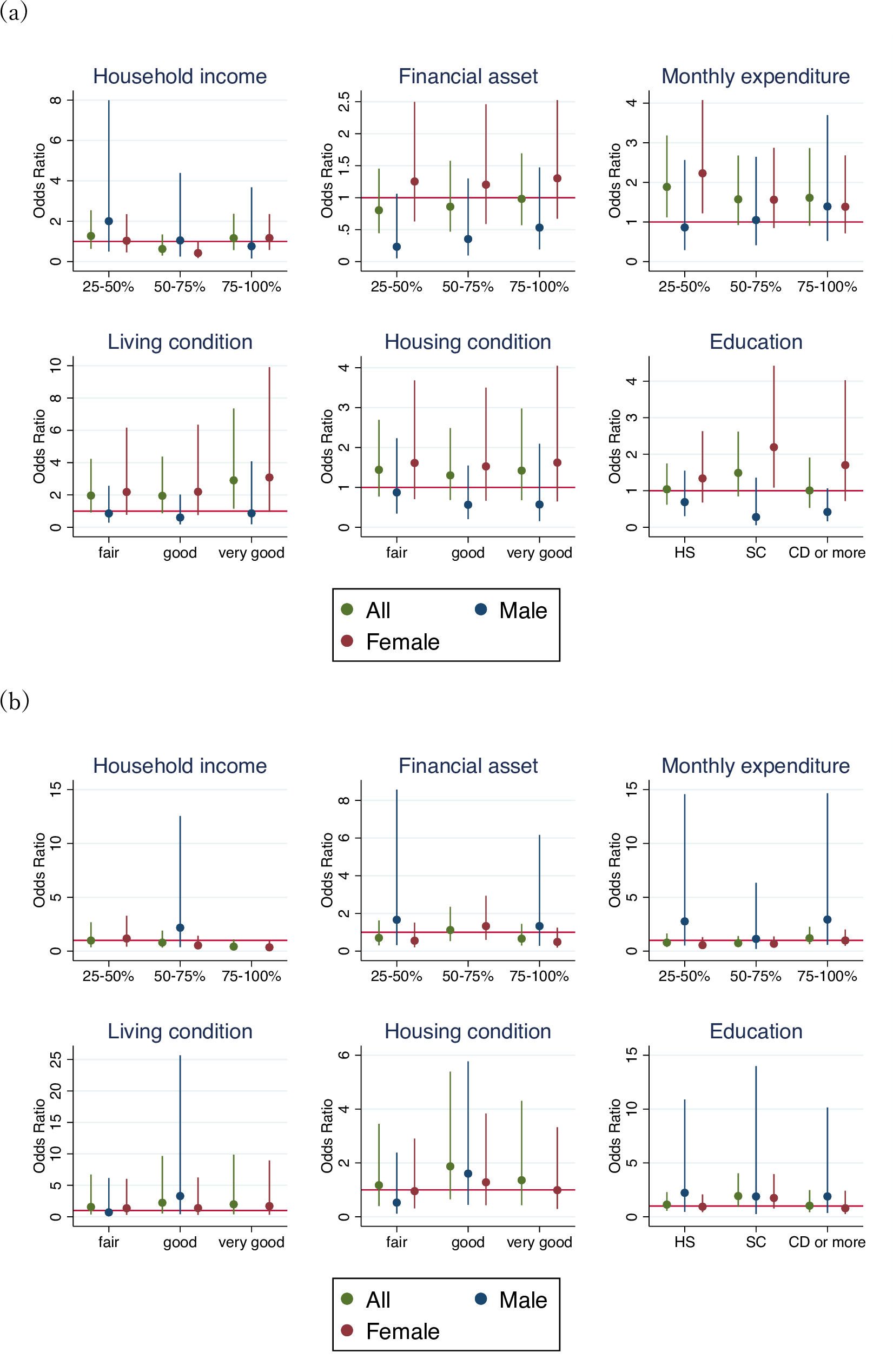

Supplement: S2 Fig — Care provision for parents by SES, own parents, or parents-in-law (a) Own parents (b) Spouse’s parents. The figure shows the odds ratio of care provision compared to the lowest SES category based on logistic regressions controlling for the age and age squared, gender (for all), marital status (for own parents, married and not widowed or divorced = 1), working status (working = 1) of respondents. The bars represent the 95% confidence interval. The dependent variable is a binary variable that takes unity if a respondent provides care to any of the parents, and zero if at least one parent is alive and the respondent does not provide care. Estimates are missing if there are no providers in the SES category. Each panel shows a different SES measure. The sample of the analysis consisted of those whose parents were alive. (a) The number of observations for all the respondents ranges from 1,708 (household income) to 3,536 (living and housing conditions). (b) The number of observations for all the respondents ranges from 985 (household income) to 2,098 (living and housing conditions). (TIF) [file pone.0256107.s002.tif]

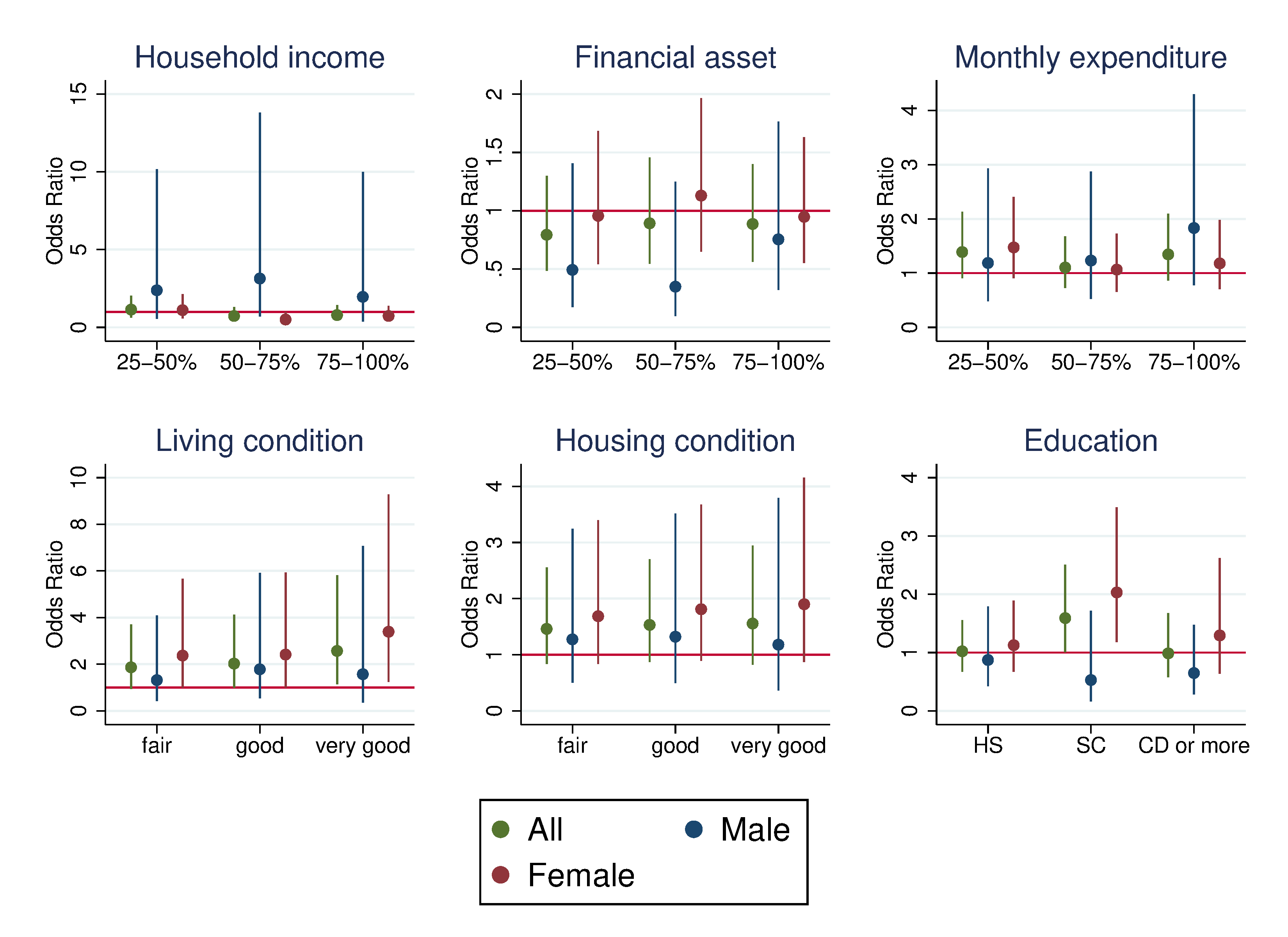

Supplement: S3 Fig — The figure shows the odds ratio of care provision compared to the lowest SES category based on logistic regressions controlling for the age and age squared, gender (for all), marital status (married and not divorced or widowed = 1), working status (working = 1), and four binary variables to show the self-rated health of the respondents. The bars represent the 95% confidence interval. The dependent variable is a binary variable that takes unity if a respondent provides care to any parent and zero if at least one parent is alive and the respondent does not provide care. Each panel shows a different SES measure. The sample of the analysis consisted of those whose parents were alive. The number of observations for all the respondents ranges from 1,708 (household income) to 3,536 (living and housing conditions). (TIF) [file pone.0256107.s003.tif]

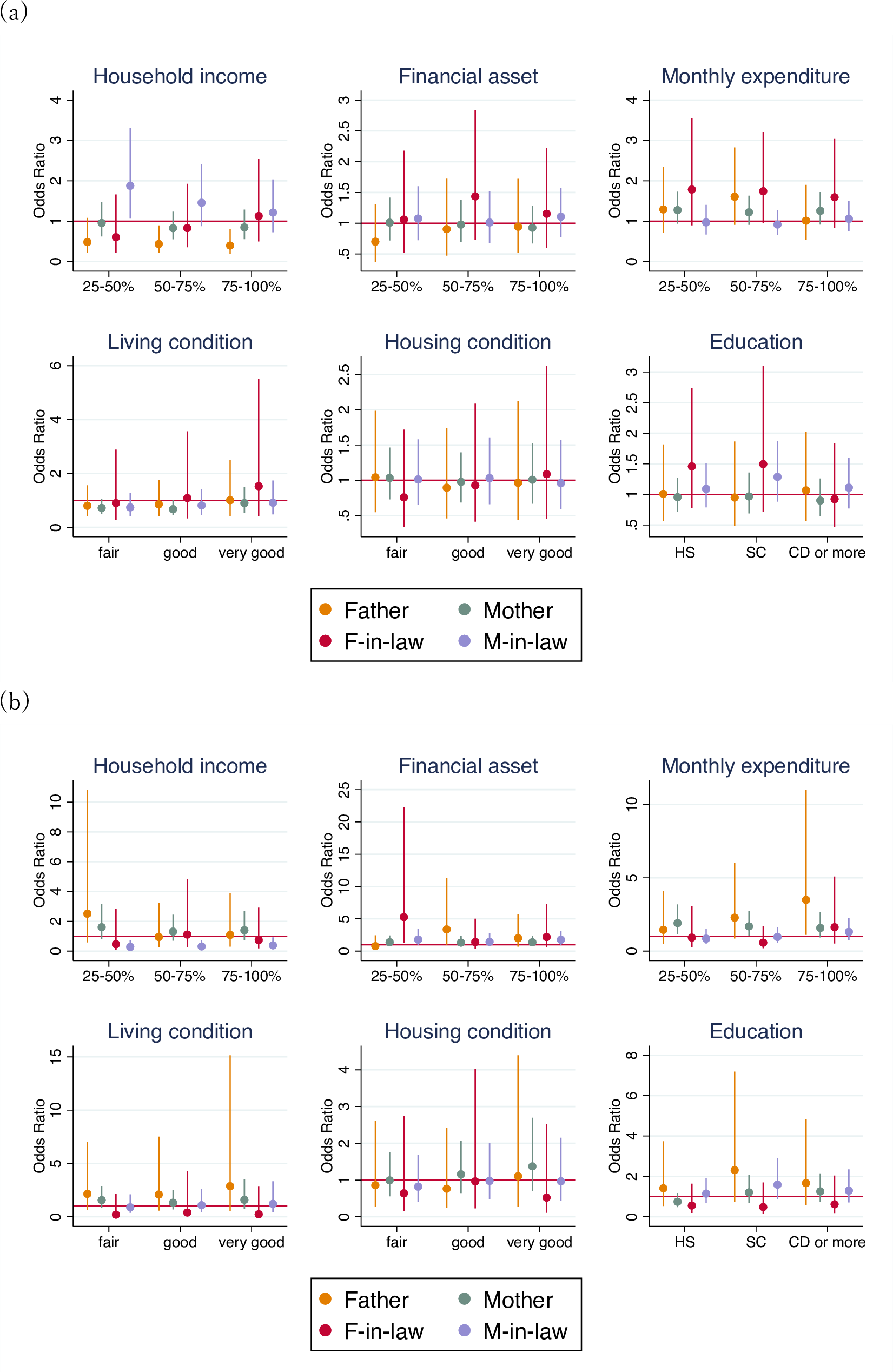

Supplement: S4 Fig — Possible mechanisms: (a) odds ratio of parents’ care needs by SES; (b) odds ratio of parents receiving care at home compared to care at a facility by SES. Panel (a) shows the odds ratio of parents receiving care at home to receiving care at a facility, based on logistic regressions controlling for the age and age squared, gender, and marital status of the respondents. The coefficient shows the association between respondents’ SES and the care needs of their parents when compared to the lowest SES category. The analysis was based on respondents whose parents were alive, and the number of observations ranges from 358 (household income for father-in-law) to 2,394 (living and housing conditions for mother). The bar represents the 95% confidence interval. In the first stage, the age of respondents and marital status (for the analysis of parents), as well as year effects, were included as independent variables, and in the second stage, only the age of parents and year effects are included. Each panel shows a different SES measure. Panel (b) shows the odds ratio of parents receiving care at home to receiving care at a facility among those who require care, based on logistic regressions controlling for the age and age squared, gender, and marital status of the respondents. The analysis was based on respondents whose parents required care, and the number of observations ranges from 81 (household income for father-in-law) to 680 (living and housing conditions for mother). The bar represents the 95% confidence interval. The dependent variable is a binary variable that takes unity if the father, mother, father-in-law, or mother-in-law requires care at home at the time of the survey, and zero if he/she receives care at a facility. Each panel shows a different SES measure. (TIF) [file pone.0256107.s004.tif]
