# Supplementary material for: Socioeconomic status and the likelihood of informal care provision in Japan: An analysis considering survival probability of care recipients
Source: PLoS One. 2021 Aug 13;16(8):e0256107. doi: 10.1371/journal.pone.0256107 (PMC8362941; doi:10.1371/journal.pone.0256107)
Supplement: S1 Table — (PDF) [file pone.0256107.s005.pdf]

S1 Table. Relationship between missing observations in household income and financial assets and self-rated health.

	Household Income		Financial Assets	
Very good		Reference		
Good	0.039*	(0.017)	0.015	(0.017)
Fair	-0.149	(0.016)	-0.010	(0.016)
Poor	-0.030	(0.020)	-0.051***	(0.020)
Very poor	-0.009	(0.034)	-0.029	(0.033)
N	7,063		7,063	
Adjusted R-squared	0.002		0.001	

Note. * p<0.5; **p<0.1; ***p<0.01. We employed a linear probability model where the dependent variable was a binary variable to indicate missing observations and the independent variables were a series of categories to show self-rated health.
