# Supplementary material for: Socioeconomic status and the likelihood of informal care provision in Japan: An analysis considering survival probability of care recipients
Source: PLoS One. 2021 Aug 13;16(8):e0256107. doi: 10.1371/journal.pone.0256107 (PMC8362941; doi:10.1371/journal.pone.0256107)
Supplement: S2 Table — (PDF) [file pone.0256107.s006.pdf]

S2 Table. Balance of SES measures with and without the inverse probability weighting

	Raw data					IPW				
	Parents are alive		Parents are not alive		p-value	Parents are alive		Parents are not alive		p-value
Household income	2.7	[1.12]	2.12	[1.08]	<0.001	2.41	[1.14]	2.44	[1.13]	0.729
Financial asset	2.39	[1.19]	2.29	[1.20]	0.005	2.3	[1.21]	2.31	[1.19]	0.77
Monthly expenditure	2.59	[1.08]	2.25	[1.04]	<0.001	2.4	[1.07]	2.44	[1.08]	0.45
Living condition	2.38	[0.71]	2.25	[0.73]	<0.001	2.32	[0.70]	2.3	[0.72]	0.57
Housing condition	2.55	[0.83]	2.38	[0.85]	<0.001	2.48	[0.83]	2.44	[0.84]	0.22
Education	2.49	[1.00]	1.95	[0.91]	<0.001	2.21	[1.00]	2.23	[1.00]	0.65

Note. IPW=inverse probability weighting. We used the inverse probability weighting to match the SES characteristics between those whose parents were alive and deceased. The mean and standard deviation (in square brackets) before and after applying the inverse probability weighting are shown. P-values show the result of the t-test for equal means between the two groups.
