# Supplementary material for: Socioeconomic status and the likelihood of informal care provision in Japan: An analysis considering survival probability of care recipients
Source: PLoS One. 2021 Aug 13;16(8):e0256107. doi: 10.1371/journal.pone.0256107 (PMC8362941; doi:10.1371/journal.pone.0256107)
Supplement: S3 Table — (PDF) [file pone.0256107.s007.pdf]

S3 Table. Summary statistics for the outcome variables

	N	Proportion
Individuals who need care (among those whose parent is alive)		
Father	858	0.204
Mother	2,397	0.284
Father of spouse	729	0.200
Mother of spouse	1,989	0.282
Individauls who receive care at home (among those who need care)		
Father	175	0.537
Mother	680	0.513
Father of spouse	146	0.479
Mother of spouse	561	0.488

Note. All the variables are the binary variables.
