# Supplementary material for: Socioeconomic status and the likelihood of informal care provision in Japan: An analysis considering survival probability of care recipients
Source: PLoS One. 2021 Aug 13;16(8):e0256107. doi: 10.1371/journal.pone.0256107 (PMC8362941; doi:10.1371/journal.pone.0256107)
Supplement: Attachment — Submitted filename: Comments for Editors on PONE-D-21-00550.pdf [file pone.0256107.s009.pdf]

Referee #?

PONE-D-21-00550

Comments on "Socioeconomic status and the likelihood of informal care provision in Japan: an analysis considering survival probability of care recipients"

1. Overall evaluation

This study aims to investigate the association socioeconomic status (SES) and the likelihood of informal care provision in Japan, using the first wave of a unique longitudinal data, entitled "Japanese Study of Aging and Retirement (JSTAR)". This study finds a new evidence that those categorized in lower SES are not necessarily more likely to provide informal long-term care (LTC) at home than those in better SES. Rather, regarding some SES measures such as self-rated living standard and female caregivers' educational achievement, even positive correlation between SES and the probability of LTC provision were reported, after adjusting "selection bias". The findings are not consistent to the results obtained by most previous studies.

The author(s) highlighted two principal contributions of this study; First one is focusing on the heterogeneity in the same choice set and finding that the heterogeneity would be unlikely to influence the probability of caregiving by informal/family caregivers; Second one is adjusting "selection bias" caused by parents' death for evaluating the association of SES and LTC care provision.

I fully agree with the contributions and novelties highlighted by the author(s). Although the author(s) did not go that far in their interpretation, their findings would have a critical policy implication, such that the economic loss in the society may be more serious than we expected, because those with higher SES and higher opportunity costs are more likely to provide informal LTC.

However, I think there are several points that need to be improved in this study. Conclusively, I recommend Editors to ask the author(s) "major revision". I would like to provide both major and minor comments as follows. Hopefully, they are helpful for the author(s) to revise this paper.

2. Comments

2-1. Missing SES at random?

As the author(s) pointed out in Discussion (page 19), "If the reporting of income and

financial assets by individuals is systematically related to SES status, it could affect the results and hence the difference across SES measures", I doubt if income and financial assets (of which number of observations are extremely few, in particular) are missing at random. So, I suggest the author(s) to conduct a test Little's test of missing totally at random.

Reference:

・Cheng Li. (2013). Little's test of missing completely at random. T*he Stata Journal* (2013)13, Number 4: 795–809.

・Little, R. J. A. (1988). A test of missing completely at random for multivariate data with missing values. *Journal of the American Statistical Association* 83: 1198–1202.

2-2. Health status of potential family caregivers

(1) This is also related to my first comment. I totally understand that this study focuses on the association of SES and the probability of providing informal care at home. Even so, since JSTAR surveys adults aged 50 and older, health status of potential family caregivers is a significant determinant of informal care provision, of which heterogeneity cannot be ignored. For example, health status of respondents could be mediator to associate the likelihood of answering questions with SES status. Similar to "selection" due to parents' mortality adjusted by this study, potential family caregivers with lower health status are less likely to provide informal care to elderly persons and their lower health status could be associated with lower or missing SES.

Fortunately, JSTAR has various indicators of respondent's health status, i.e. self-rated health status (SRH) and type of disease. Therefore, I would like to suggest the author(s) to test whether or not if a representative health status like SRH is statistically significantly correlated with the probability of missing SES.

(2) Further, regarding health status of potential family caregivers, I would like to suggest the author(s) to investigate the association of a representative indicator like SRH and the likelihood of LTC provision like SES measures. Or, the author(s) can include SRH as a control variable into regression analyses.

2-3. Heterogeneity in regions and timings of the survey

(1) This study utilizes the first wave of JSTAR datasets which surveyed 10 different municipalities and was conducted in various years, 2007, 2009, and 2011. First, the

characteristics of these municipalities vary in terms of the size of population, the age structure, the fiscal scale, the geographic area, the supply of medical care and LTC, etc. I think the author(s) should include municipality dummies as covariates for adjusting these characteristics if the data are available, as these geographical characteristics might be correlated to the accessibility to formal LTC services, which would have a significant impact on the probability of demand for informal care by family members.

(2) Second, like geographical heterogeneity, the timings of the survey also vary. Although the author(s) confirmed in Data and Methods (page8), "we observed few changes in SES and care provision over time", I still think that year dummies should include in regression analyses. For example, the global macro economy experienced Bankruptcy of Lehman Brothers in 2009 and we had the Great East Japan Earthquake in 2011, which would be influence people's SES and thus various household-level decision makings. Further, I also suggest to include interactive terms of municipality and year dummies, because the impacts of these drastic events would vary across regions.

2-4. Japan's LTC insurance

In order to discuss the findings, the author(s) explained the background of public LTC insurance briefly in Discussion section (p17-18). However, I think it would be helpful for international readers to understand the context of this study if the author(s) provide a brief summary for the background and system of Japan's LTC insurance system in an additional section.

2-5. Policy implications

As I pointed out in the overall evaluation, I think this study would have a critical policy implication, such that the economic loss in the society may be more serious than we expected, because those with higher SES and higher opportunity costs are more likely to provide informal LTC. Since this study aims to describe the "correlation" between SES status and the likelihood of informal LTC provision, rather than identifying any causal relations, it is understandable why the author(s) did not go that far to interpret their findings. However, at least, the author(s) had better to mention what might happen to potential socio-economic surplus and/or social welfare if their findings are true.

2-6. Minor comments

(1) Dependent variables explained in page 11 ("the variable takes unity if the parent received home-based or residential care, and zero if the parent does not require care)

are not shown in basic statistics (Table 1). The author(s) had better to show these, although they are not major dependent variables.

(2) Is the following sentence in page 12 necessarily described? “Income and expenditure could be measured at the individual level”. I think the author(s) have already explained this in the former section and this is a kind of redundant.

(3) In Table 1 (page 30), please clarify the numbers of observations (N) for outcome measures. They vary among “to any of the parents”, “to own parents”, and “to spousal parents”, because the author(s) extracted only those whose parents are alive? I might overlook the clarification in the manuscript. But, to me, it is unclear how to determine the number of samples to be regressed.

(4) Related to the above comment (3), please clarify the numbers of observations to be analyzed in all figures including the ones in Appendix. For example, are all the number of observations the same among different SES measures in Figure1 and 2? As long as I see Table 1, the number of available observations vary across these SES measures and so they must be different in Figure 1 & 2.

(5) Also, I am not clear if the author(s) run the regression including these SES variables simultaneously in the same regression estimation. Or, they put each of them in separate regression. Please specify in “Methods”.

(6) Regarding Table A1 in Appendix, please show test statistics how significantly statistically different the mean of each SES measure between two groups (parent are alive versus parents are not alive) is.
