# Supplementary material for: Socioeconomic status and the likelihood of informal care provision in Japan: An analysis considering survival probability of care recipients
Source: PLoS One. 2021 Aug 13;16(8):e0256107. doi: 10.1371/journal.pone.0256107 (PMC8362941; doi:10.1371/journal.pone.0256107)
Supplement: Attachment — Submitted filename: 210623ResponseLetter.docx [file pone.0256107.s011.docx]

**Response to comments from the editor and reviewers**

(Comments from the editor and reviewers *in italic black* and **authors’ responses in bold blue font)**

**We are grateful for the valuable comments provided by the editor and the two reviewers. We believe that incorporating these comments has helped us to improve the quality of the paper. We hereby provide our detailed responses to each comment below. We have also made minor revisions for better readability and these changes are tracked.**

*Reviewers' comments: 
Reviewer 1:*

*The authors revised the manuscript to meet my previous comments. However, the following concern remain, which should be addressed.

1. Discussion part, p.22, l.11-12, Conclusion p.26, l.14-15
The authors mentioned “we did not find strong evidence of a higher burden of caregiving falling on individuals with lower SES.” in the discussion part.
However, the authors reached the conclusion that says “Although a negative association between SES and care burden has been repeatedly reported in terms of care intensity, it is important to note that the decision around caregiving could differ in relation to SES”.*

**We apologize for the unclear explanation. We revised the first sentence as follows:**

**“Unlike studies that examined the burden of caregiving among caregivers by SES, we did not find strong evidence of a higher burden of caregiving falling on individuals with lower SES in terms of a decision on whether one provides informal care.”**

*Reviewer 2:*

*The author(s) clarified all my questions and revised following my comments as possible as they can. However, the limitation of the data seems to be quite serious that the author(s) should clarify the further analyses to be necessary.*

**Thank you for pointing this out. We expanded the limitation according to reviewer’s comments. Also, we stated that further research is necessary in the abstract.**
